# Flame Retardancy, Thermal and Mechanical Properties of Novel Intumescent Flame Retardant/MXene/Poly(Vinyl Alcohol) Nanocomposites

**DOI:** 10.3390/nano12030477

**Published:** 2022-01-29

**Authors:** Xiaofei Yan, Jie Fang, Jianjun Gu, Chenkai Zhu, Dongming Qi

**Affiliations:** 1 Zhejiang Provincial Engineering Research Center for Green and Low-carbon Dyeing & Finishing, Zhejiang Sci-Tech University, Hangzhou 310018, China; 202030202089@mails.zstu.edu.cn; 2 Key Laboratory of Advanced Textile Materials and Manufacturing Technology, Ministry of Education, Zhejiang Sci-Tech University, Hangzhou 310018, China; 201930306041@mails.zstu.edu.cn; 3 Zhejiang Provincial Key Laboratory of Fiber Materials and Manufacturing Technology, Zhejiang Sci-Tech University, Hangzhou 310018, China; 4 College of Textile Science and Engineering (International Institute of Silk), Zhejiang Sci-Tech University, Hangzhou 310018, China; 5 Ningbo Institute of Technology, Beihang University, Ningbo 315832, China; chenkaizhu@zstu.edu.cn

**Keywords:** flame-retardant properties, MXene/PVA nanocomposites, poly(vinylphosphonic acid), polyethylenepolyamine

## Abstract

Poly(vinylphosphonic acid) (PVPA) and polyethylenepolyamine (PEPA) are used as novel intumescent flame retardants to improve the properties of MXene (2D Ti_3_C_2_T*_x_*)/poly(vinyl alcohol) (PVA) nanocomposites. We investigated the flame-retardant properties, thermal stability, and mechanical properties of MXene/PVA nanocomposites. The results show that MXene was homogeneously dispersed in the PVA matrix containing PVPA and PEPA. PVPA and PEPA effectively improved the flame-retardant properties of MXene/PVA nanocomposites and they did not obviously change the thermal degradation of the MXene/PVA nanocomposites. Moreover, MXene improved the thermal stability of the PVA matrix. The elongation at break of MXene/PVA nanocomposites reached its maximum when the MXene mass fraction was 1.0 wt.%, regardless of whether PVPA and PEPA were present in the PVA matrix, whereas the tensile strength and Young’s modulus of MXene/PVA nanocomposites increased with the increase in MXene content in the PVA matrix.

## 1. Introduction

Poly(vinyl alcohol) (PVA) is an excellent material due to its water solubility, dopant-dependent electrical and optical conductivity [1], thermostability [2], and easy processability [3], and is widely applied in fibers [4], films [5], and membranes [6]. However, insufficient mechanical and flammability properties of PVA have restricted its wider applications in some fields [7]. Many nano-inorganic additives, such as silicate clay, carbon nanotubes, ultrathin hydroxide, phenyl-phosphonate, layered double hydroxide, α-ZrP, graphene oxide, graphene, and MoS_2_, were introduced to extend the application of PVA [8]. However, one of the fatal defects of inorganic/PVA nanocomposites is easy flammability under fire when applied as fibers and adhesive for construction.

At present, halogen-free flame retardants have attracted increasing attention in the field of flame retardants [9]. It is reported that flame retardants containing P, N, and Si elements are more environmentally friendly and effective for polymers [10]. Intumescent flame retardants such as poly(vinylphosphonic acid) (PVPA) and polyethylenepolyamine (PEPA) are halogen-free flame retardants with efficacious P and N flame retardants that are widely used in improving the flame retardance of polymers due to their excellent thermal stability, low cost, low smoke, and toxicity [11]. Opwis et al. [12] found a thin coating of PVPA provided an effective barrier to fire when applied to the surfaces of glass-fiber-reinforced epoxy resin composites. Williams et al. [13] demonstrated that PVPA was effective in fire-retarding a typical meltable and flammable poly(methyl methacrylate). In addition, Li [14] studied the effects of surface treatment of carbon fiber (CF) by PEPA on the interfacial adhesion behavior and the flame retardancy of polypropylene/polystyrene matrix blends filled with CF composites, and the composites presented good flame-retardant properties. However, such studies on the polymer-based composites containing PVPA and PEPA together as the flame retardant are rare, not to mention the synergistic flame-retardant effect with other inorganic flame retardants.

Transition metal carbides and nitrides (Ti_3_C_2_T*_x_*, also known as MXene) are one of the most recent 2D inorganic nanomaterials with unique properties that have been widely applied in saturable absorbers [15], nonlinear photonics [16], energy storage [17], conductive electrodes [18], field-effect transistors [19], and biomedicine [20], exhibiting outstanding performances. It has robust hydrophilicity and hydroxyl groups which can be compatible with soluble polar polymers, especially PVA. Studies related to MXene/PVA nanocomposites have been investigated by many researchers [21,22,23,24,25]. Ling et al. [22] found that the MXene/PVA nanocomposite can withstand 15000 times its own weight without visible deformation or damage, and MXene can also improve the mechanical strength over three times higher than that of PVA. Sobolčiak et al. [23] studied that the PVA nanofibers containing 0.14 wt.% cellulose nanocrystals (CNC) exhibited an increase in the storage modulus of pure PVA nanofibers by 20% while the storage modulus increased by more than 100% after the addition of 0.07 wt.% MXene nanosheets and 0.07 wt.% CNC. Liu and Li [21] fabricated MXene/PVA composite film with an MXene weight percent as high as 87.29% and found the formation of Ti–O bonds increased the thermal properties of both MXene and PVA. Xu et al. [24] reported the MXene/PVA composite foams achieved excellent absorption–dominated shielding performance of 28 dB with only 0.15 wt.% of MXene. Yu et al. [25] discovered that adding 2.0 wt.% MXene to polyurethane could improve flame-retardant properties by reducing smoke product release and toxicity hazards. However, very little research on the flame retardancy of the MXene/PVA nanocomposites containing PVPA and PEPA has been performed.

This research primarily discusses the flame-retardant properties and the mechanism of intumescent flame retardant (PVPA and PEPA)/MXene/PVA nanocomposites in detail. The dispersion status of MXene in the PVA matrix containing PVPA and PEPA was analyzed firstly, and the thermal stability and the mechanical properties of the nanocomposites were also investigated.

## 2. Materials and Methods

### 2.1. Materials

Poly(vinyl alcohol) (PVA) was purchased from Shanghai Chenqi Chemical Technology Co., Ltd., Shanghai, China, which has a molecular weight range from 118,000 to 124,000 and a polymerization degree of 2450 ± 50. Ti_3_AlC_2_ (MAX) powder was obtained from Nanjing Mingchang New Material Technology Co., Ltd., Nanjing, China. Concentrated hydrochloric acid (HCl), lithium fluoride (LiF), vinyl phosphonic acid (VPA), sodium hydroxide (NaOH, 98%), 2,2′-azobis(isobutyramidine) dihydrochloride (AIBA, 98%), and polyethylenepolyamine (PEPA) were supplied by Shanghai Aladdin Biochemical Technology Co., Ltd., Shanghai, China. All materials were obtained from commercial sources and were used without further purification.

### 2.2. Preparation of the Specimens

#### 2.2.1. Synthesis of MXene (Ti_3_C_2_T_*x*_) Nanosheets

MXene was prepared following the literature [26]. One gram of LiF was added into 20 mL of 9 mol/L HCl solution and the mixture was stirred for 10 min at 40 °C. Then, 1 g of Ti_3_AlC_2_ was slowly added into the mixed solution, which was continuously stirred for 24 h at 40 °C. The obtained reaction suspension was washed with distilled water several times until the pH of the suspension, was above 6. It was sonicated by an ultrasonic cleaner (KQ-250DE, Kunshan Ultrasonic Instrument Co., Ltd., Suzhou, China) with 40 kHz at 250 W for 30 min after 50 mL deionized water was added to the suspension which was operated under the protection of an ice-water bath and argon atmosphere. The suspension was centrifuged to separate the powder, and the above product was filtered under vacuum conditions and dried at room temperature for 24 h to obtain the Ti_3_C_2_T*_x_* (MXene) nanosheets.

#### 2.2.2. Preparation of Poly(vinylphosphonic Acid)

According to the literature [27], 81.2 g of VPA and 100 mL of distilled water were added to a dry 100 mL three-necked flask equipped with magnetic stirring and mixed with 15.0 g of NaOH in an ice bath. Then, 1.52 g of AIBA was added to the resulting solution, which was placed in a Schlenk flask and degassed by bubbling with nitrogen for 40 min. Finally, the Schlenk flask was evacuated and backfilled with nitrogen. The reaction mixture was heated to 65 °C for 8 h to obtain PVPA.

#### 2.2.3. Preparation of MXene/PVA Nanocomposites

The MXene/PVA nanocomposites were fabricated by the solution blending method. The MXene nanosheets, PVPA and PEPA were dispersed in deionized water and under ultrasound for 1 h to form a uniform suspension, respectively, while the PVA was dissolved in deionized water at 85 °C to form the aqueous solution. The obtained MXene nanosheets, PVPA and PEPA solutions were added dropwise to the PVA aqueous solution during stirring and ultrasonication for 1 h to form the MXene/PVA nanocomposites stock solution, respectively. The stock solution was poured into a mold with dimensions of 100.0 mm × 100.0 mm × 5.0 mm and placed in an oven at 35 °C and 60% relative humidity for 1 day. The film was demolded and dried at 60 °C for 1 h to get the MXene/PVA nanocomposites containing PVPA and PEPA, and the detailed formulations of PVA nanocomposites are presented in Table 1.

### 2.3. Methods

#### 2.3.1. Morphology and Elemental Analysis

An ultrahigh-resolution field emission scanning electron microscopy (SEM, Zeiss GeminiSEM 500, Carl Zeiss AG, Oberkochen, Germany) was used to observe the dispersion of MXene, PVPA and PEPA in PVA matrix after treated by the liquid nitrogen, and the char residue after the samples fully burned. Transmission electron microscopy (TEM) images were operated on a Hitachi model H-800 TEM (Tokyo, Japan) with an accelerating voltage of 200 kV to observe the morphology of delaminated MXene. To measure the average lateral size of MXene, SEM photos of the nanosheets were taken, and Adobe Photoshop CC 2017 and ImageJ were then used to determine lateral size of MXene nanosheets according to the scale of SEM nanosheets photos. At least 500 nanosheets (about six photos) were examined, and the measurements were statistically analyzed. The specimens which were treated with the liquid nitrogen and residues were analyzed by the SEM coupled with energy dispersive X-ray (EDX). The surface elements were attained from EDX (0.2–20 keV) on an EMAX energy spectroscopy (HORIBA, Ltd., Kyoto, Japan). The calibration method for EDX analysis was operated according to ISO 29081-2010. All samples were coated with gold before SEM testing was operated with a primary electron beam at an accelerating voltage of 2 kV.

#### 2.3.2. Flame-Retardant Properties Measurement

The limiting oxygen index (LOI) denotes the lowest volume concentration of oxygen sustaining a candle burning materials in mixed gases of nitrogen and oxygen. It was conducted according to ISO4589-1984 standard by JF-5 automatic oxygen index analyzer (Nanjing Jionglei Instrument Equipment Co., Ltd., Nanjing, China) and the dimensions of all the samples were 140 mm × 50 mm × 5 mm.

The vertical burning test was operated by a vertical burning test instrument (CZF-2, Jiangning Analysis Instrument Factory, Nanjing, China) according to the American National Standard UL-94 with the size of a sample of 125 × 12.5 × 5.0 mm^3^ (5.0 mm thickness). The UL-94 test results are classified by burning ratings V-0, V-1, or V-2, V-0 which implies whether that the specimen has good flame-retardant properties.

The heat release rate (HRR), the time to ignition (TTI), total heat release (THR), peak-heat release rate (pHRR), and total smoke production (TSP) were recorded by the cone calorimeter (Fire Testing Technology Limited, East Grinstead, West Sussex, UK) tests according to ISO 5660-1-2015 standard. Five samples were repeated for each measurement. Each specimen, with dimensions of 100 mm × 100 mm × 5 mm, was packaged in aluminum foil and radiated horizontally to an external heat flux of 35 kW/m^2^. The fire performance index is recognized to be a reliable indicator of a polymer’s flammability [28] which is shown in Equation (1).

(1) $$\mathrm{FPI}=\frac{t_{ign}}{\mathrm{pHRR}}$$

where, $t_{ign}$ is the time to ignition; $t_{\mathrm{pHRR}}$ is the time to pHRR.

In general, the higher the FPI value, the higher the flame-retardant properties of the material.

#### 2.3.3. Thermal Stability Properties Measurement

Thermal gravimetric analysis (TGA) was performed on a Netzsch 209F1 thermogravimetric analyzer (Selb, Bavaria, Germany) at a heating rate of 10 °C/min. The samples were tested under nitrogen atmosphere with a follow rate of 40 mL/min at temperature range from 30 °C to 600 °C. Samples of 5–10 mg were heated. All the thermal degradation data were obtained from the TG and DTG curves.

Differential scanning calorimeter (DSC) measurement was carried out with a TA Q800 instrument (TA Instruments Inc., New Castle, DE, USA). Five-to-ten milligrams of specimen was heated from 40 °C to 600 °C at a heating rate of 10 °C/min under a constant nitrogen flow of 40 mL/min.

TGA-FTIR (Fourier transform infrared) tests were recorded with a thermal gravimetric analyzer (Q50, TA Instruments Inc., New Castle, DE, USA) which was connected to a Nicolet IS50 spectrometer (Thermo Fisher Scientific Inc., Waltham, MA, USA), 5–10 mg samples were tested under a nitrogen atmosphere from 30 °C to 600 °C with a heating rate of 10 °C/min.

#### 2.3.4. Mechanical Properties Measurement

Mechanical properties of the samples were tested on a universal testing machine (3369, Instron Co., Ltd., Norwood, MA, USA) at constant strain rate 2 mm/min in accordance with ASTM D638. The samples were cut into strips (50 mm × 4 mm × 0.5 mm) and tested with a gauge length of 40 mm, using five samples for each measurement.

## 3. Results and Discussion

### 3.1. Characterization MXene and the Dispersion of MXene in the Nanocomposites

**Figure 1:**
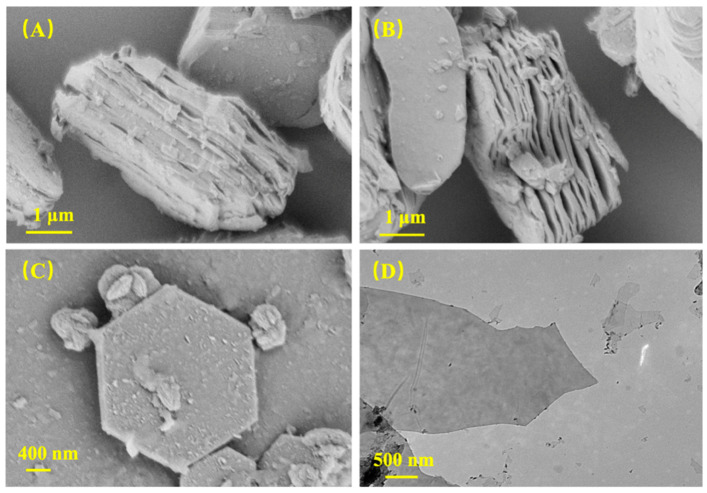
Images of MXene and MXene/PVA nanocomposites: SEM images of etched MXene (**A**,**B**); and delaminated MXene (**C**); TEM image of delaminated MXene (**D**).

The SEM were applied to characterize the morphology of the prepared MXene nanosheets and the dispersion of MXene, PVPA, and PEPA in PVA the matrix. Figure 1 presents the variation SEM and TEM images of MXene before and after delamination and MXene/PVA nanocomposites, respectively. It can be seen from Figure 1A,B that the multi-layered stacked MXene was obtained after selectively etching the Al from a MAX powder by using LiF and HCl. The single-layered MXene had a typical hexagonal symmetry which was acquired by sonicating the multi-layered stacked MXene suspension, as shown in Figure 1C. The MXene nanosheets have an average lateral size of 701.0 nm. In addition, the TEM of the MXene nanosheets are illustrated in Figure 1D, and corresponded to a single layer of the MXene nanosheets, implying the successful exfoliation of the nanosheets.

**Figure 2:**
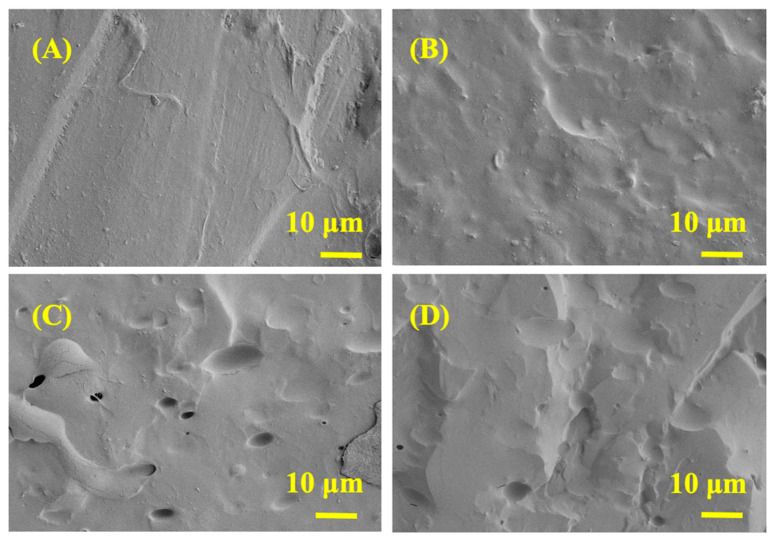
SEM and element-mapping images of PVA and MXene/PVA nanocomposites after brittle fracture: (**A**) PVA; (**B**) MXene_1.0%_/PVA; (**C**) PVA-PVPA_10%_-PEPA_5%_; and (**D**) MXene_1.0%_/PVA-PVPA_10%_-PEPA_5%_.

**Figure 3:**
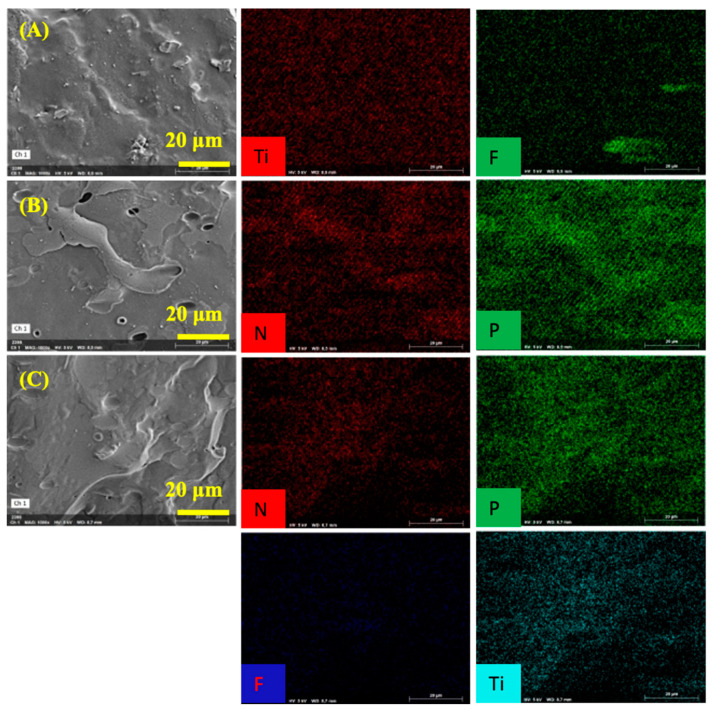
SEM and element-mapping images of PVA and MXene/PVA nanocomposites after brittle fracture: (**A**) MXene_1.0%_/PVA; (**B**) PVA-PVPA_10%_-PEPA_5%_; and (**C**) MXene_1.0%_/PVA-PVPA_10%_-PEPA_5%_.

The dispersion of MXene, PVPA, and PEPA in PVA matrix were observed by SEM and element-mapping images which were shown in Figure 2 and Figure 3, respectively. It can be also obtained from Figure 2B and Figure 3A that MXene nanosheets have excellent dispersibility in PVA, which also means that the MXene nanosheets can be easily introduced into PVA aqueous solution. Figure 2C,D and Figure 3B,C prove that PVPA and PEPA can be also homogeneously dispersed in the PVA matrix. In addition, there is no obvious aggregation of MXene in the PVA matrix with PVPA and PEPA, as shown in Figure 2D.

### 3.2. Flame-Retardant Properties

The LOI and UL94 test results of the MXene/PVA nanocomposites with different contents of PVPA and PEPA are shown in Table 2. The MXene had a positive effect on the flame-retardant property of PVA, and the MXene/PVA nanocomposite with only 1.0 wt.% of MXene presented the highest LOI value among all the MXene/PVA nanocomposites without any flame retardant. The LOI value of MXene_1.0%_/PVA nanocomposite increased 10.5% when compared with that of the pure PVA. However, the LOI value of MXene_0.5%_/PVA increased only fractionally, and the UL 94 rating was the same with pure PVA. In addition, flaming melt drop was observed in the MXene/PVA nanocomposites without PVPA and PEPA.

Moreover, the LOI value and UL 94 rating of PVA containing both PVPA and PEPA exhibited much better performance than that of pure PVA, indicating that PVPA and PEPA have a positive effect on the flame-retardant properties of the pure PVA. The LOI value of PVA-PVPA_10%_-PEPA_5%_ can be as high as 25.50%, which has been dramatically increased by 34.2% compared to the pure PVA.

**Figure 4:**
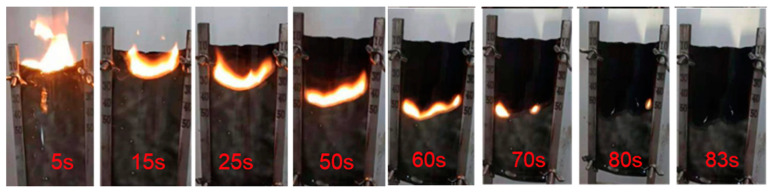
The burning behaviors of MXene_1.0%_/PVA-PVPA_10%_-PEPA_5%_ during the LOI value = 27.50%.

All the MXene/PVA nanocomposites containing both PVPA and PEPA demonstrated good LOI values and UL 94 rating. This established that the PVPA and PEPA can significantly improve the LOI values and UL 94 rating of MXene/PVA nanocomposites. The LOI value of MXene/PVA nanocomposite containing both PVPA and PEPA reached the peak (27.50%) when the mass fraction of MXene was 1.0 wt.%. The MXene_1.0%_/PVA-PVPA_10%_-PEPA_5%_ nanocomposite exposed a good self-extinguishment, the smoke emission during the whole burning process was small, and non-flaming melt drop was observed as shown in Figure 4.

**Figure 5:**
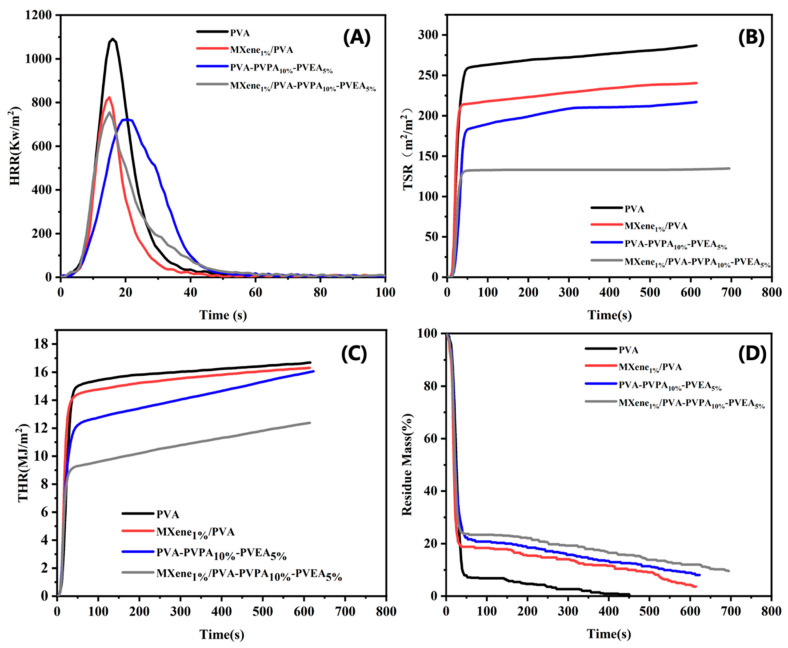
HRR (**A**), THR (**B**), TSR (**C**), Residue (**D**) curves of PVA and MXene/PVA nanocomposites.

The corresponding flame-retardant performance parameters, including the time to ignition (TTI), peak-heat release rate (pHRR), total heat release (THR), total smoke release (TSR), fire performance index (FPI = TTI/PHRR), and residue for cone calorimeter test are listed in Table 3, and the measured heat release rates (HRR), THR, TSR and residue mass curves of all the composite samples at 35 kW/m^2^ are presented in Figure 5. It can be obtained from Table 3 and Figure 5 that the MXene, PVPA, and PEPA had a positive effect on the flame-retardant properties of PVA matrix. MXene demonstrated outstanding performance on flame-retardance and smoke suppression. When compared to pure PVA, the PHRR and THR of MXene_1.0%_/PVA were 823.9 kW/m^2^ and 16.47 MJ/m^2^, respectively, which were reduced by 24.5% and 1.3%, respectively. In addition, the PVA-PVPA_10%_-PEPA_5%_ has a large decline in the pHRR and THR compared to the PVA. In addition, it is found that the downward trends of the pHRR and THR were even more pronounced when the PVPA and PEPA were together in the MXene/PVA nanocomposite, and it also proved the good flame-retardant property of MXene_1.0%_/PVA-PVPA_10%_-PEPA_5%_.

The FPI of MXene_1.0%_/PVA (0.012 s·m^2^/ kW) was found to be higher than that of neat PVA (0.008 s·m^2^/ kW), whereas the FPI of MXene_1.0%_/PVA-PVPA_10%_-PEPA_5%_ was found to be the highest among all the samples, implying the flame retardant effects of PVPA and PEPA on the MXene/PVA nanocomposite. Moreover, the TTI of PVA and MXene/PVA nanocomposites had similar trends to FPI.

**Figure 6:**
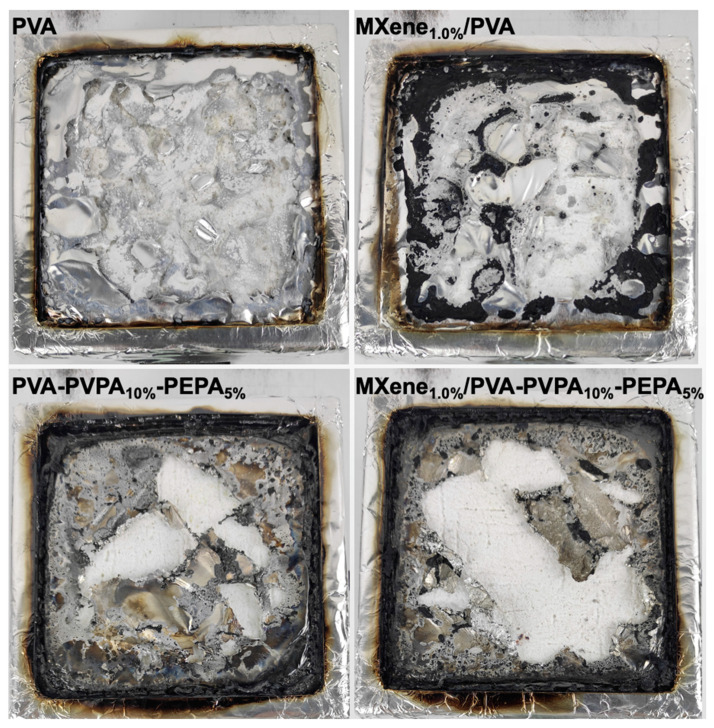
Char residue of PVA and MXene/PVA nanocomposites after cone calorimeter test.

The TSR value of PVA decreased from 287.48 m^2^/m^2^ to 240.37 m^2^/m^2^ compared to that of MXene_1.0%_/PVA. Additionally, 1.0 wt.% of Ti_3_C_2_T_*x*_ was added to the PVA-PVPA_10%_-PEPA_5%_ has improved the TSR value from 216.89 m^2^/m^2^ to 134.61 m^2^/m^2^. Because of the catalytic and barrier effects, MXene demonstrated excellent smoke suppression effects. The addition of PVPA and PEPA together dramatically decreased the TSR value of pure PVA and MXene/PVA nanocomposite, respectively. The residual mass of the samples after the cone calorimeter test grew when the amount of additive was also increased in the formula of PVA nanocomposites. The photographs of char residues are shown in Figure 6.

**Figure 7:**
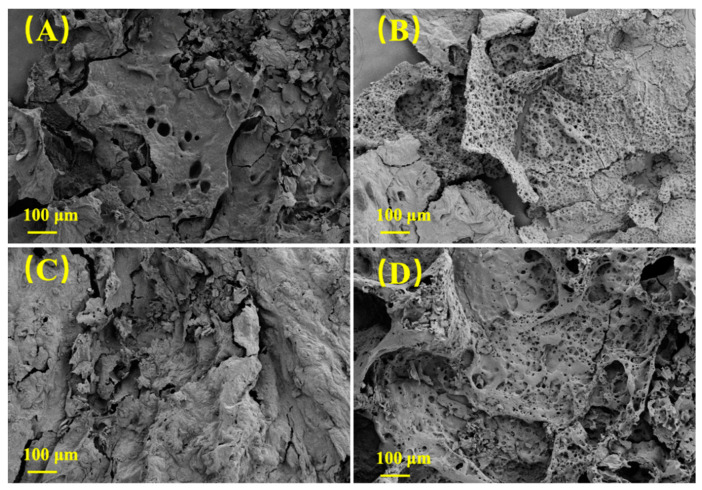
SEM of char residue of PVA and MXene/PVA nanocomposites after cone calorimeter test: (**A**) PVA; (**B**) MXene_1.0%_/PVA; (**C**) PVA-PVPA_10%_-PEPA_5%_; and (**D**) MXene_1.0%_/PVA-PVPA_10%_-PEPA_5%_.

The char residue microstructure of PVA and MXene/PVA nanocomposites after cone calorimeter test was observed by SEM, as illustrated in Figure 7. It was observed that there were a lot of micropores on the outer surface of MXene_1.0%_/PVA and MXene_1.0%_/PVA-PVPA_10%_-PEPA_5%_, indicating a large amount of heat transmission and fuel gas diffusion. MXene was oxidized into TiO_2_ [29,30], which covered the surface of the char layer, isolating the air to achieve a good flame-retardant property [31]. It can be also seen from Figure 7 that the pure PVA or MXene/PVA nanocomposite containing PVPA and PEPA was more compact and intact than that of the pure PVA. All the results imply that the char layer of MXene_1.0%_/PVA-PVPA_10%_-PEPA_5%_ reduced the heat transmission from outside and the fuel gas diffusion from inside. This was attributed to the compound effect of the flame retardant between MXene, PVPA, and PEPA in the formation of a uniformly covered, non-permeable surface char layer.

**Figure 8:**
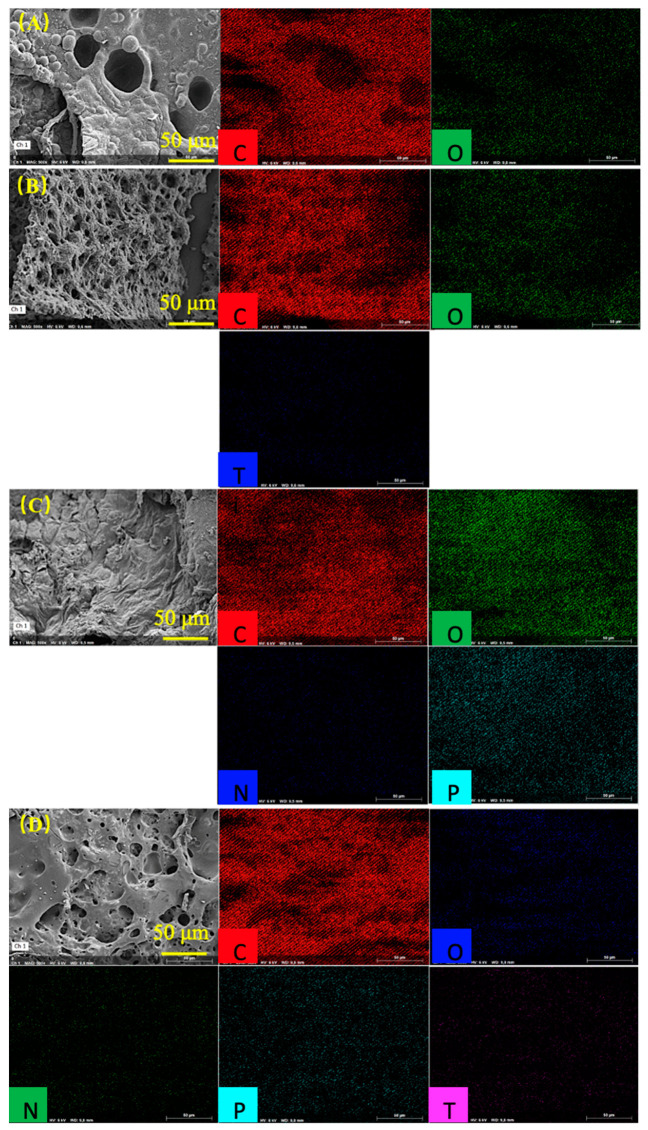
SEM and element-mapping images of char residue of PVA and MXene/PVA nanocomposites after cone calorimeter test: (**A**) PVA; (**B**) MXene_1.0%_/PVA; (**C**) PVA-PVPA_10%_-PEPA_5%_; and (**D**) MXene_1.0%_/PVA-PVPA_10%_-PEPA_5%_.

The enlarged SEM and element-mapping images of PVA and MXene/PVA nanocomposites after cone calorimeter test were presented in Figure 8. It can also be clearly seen from Figure 8 that the micropores on the outer surface of MXene_1.0%_/PVA are much more uniform and denser than those of MXene_1.0%_/PVA-PVPA_10%_-PEPA_5%_, and it was mainly caused by all the nitrogen elements in PEPA of MXene_1.0%_/PVA-PVPA_10%_-PEPA_5%_ being oxidized to gaseous nitrogen-containing oxides under the action of TiO_2_ as the catalyst [30], which came from the oxidized MXene, and a lot of the gaseous nitrogen-containing oxides were released immediately from the inside of the char residue. The char residue of PVA containing PVPA and PEPA looked more complete, and the cracks were smaller than those of the PVA, implying that metaphosphoric acid was generated and covered the char surface to isolate oxygen during the burning process of P elements in PVPA. In addition, some of the -NH_2_ and N elements in the PEPA were oxidized after absorbing the heat released during the combustion process, and all those made the heat released during the burning process smaller than that of PVA.

The concentrations of C, O, Ti, P, and N in the char residues of the PVA, MXene_1.0%_/PVA, PVA-PVPA_10%_-PEPA_5%_, and MXene_1.0%_/PVA-PVPA_10%_-PEPA_5%_ after cone calorimeter test were listed in Table 4. For MXene_1.0%_/PVA, the atom percent of Ti in the char residues was 7.60%, resulting from the TiO_2_ accumulated or pushed by the volatile products onto the surface during the thermal degradation of MXene in PVA. Moreover, for PVA-PVPA_10%_-PEPA_5%_, the atom percentages of P and N in the char residue were 6.11% and 5.00%, respectively. The total fire-retardant elements were enriched in the char residue, and it is the main reason that the fire resistance property of pure PVA with PVPA and PEPA is better than that of pure PVA and MXene_1.0%_/PVA.

The atom percentages of Ti and P in the char residue for MXene_1.0%_/PVA-PVPA_10%_-PEPA_5%_ were 4.60% and 3.02%, respectively. It formed a stabilized char layer containing the titanium–phosphate structure due to the reactions between the titanium and phosphorous elements. However, all the N in the char residues was oxidized to gaseous nitrogen-containing oxides under the effect of Ti_3_C_2_T*_x_* and PVPA, and the process of N oxidation absorbed lots of heat which made the temperature of the char surface lower. In addition, the C/O ratio of MXene_1.0%_/PVA-PVPA_10%_-PEPA_5%_ was 8.29 which is the highest among all the specimens in Table 4. This meant that an aromatic structure formed on the surface of char residue, which was beneficial for improving the self-extinguishment and flame inhibition of MXene/PVA nanocomposites during burning.

The char layer on the surface of the polymer enhanced the physical barrier effect, preventing the flammable gases, heat flux, and flame from the polymer matrix of the sublayer. When PEPA and PVPA were exposed to fire, thermo-oxidatively degraded to give a highly intumescent carbon-rich char layer which coated the surface of the nanocomposites. This expanded char layer substantially delayed the ignition (or in some cases, prevented ignition) of the underlying polymer, leading to much lower peak rates of heat release. All of these factors contributed to MXene1.0%/PVA-PVPA10%-PEPA5% having the best flame-retardant property.

### 3.3. Thermal Properties

**Figure 9:**
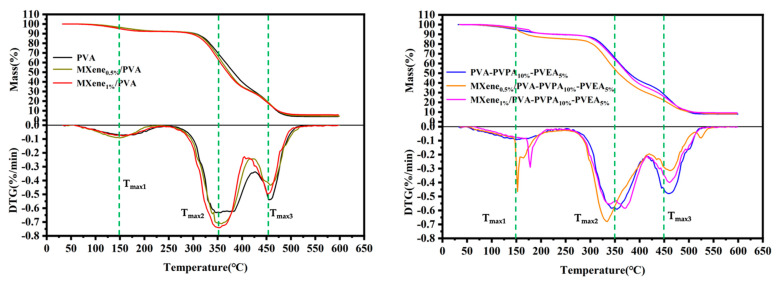
TG and DTG curves of the PVA and MXene/PVA nanocomposites under nitrogen atmosphere.

The TGA curves of PVA and MXene/PVA nanocomposites under a nitrogen atmosphere are exhibited in Figure 9 while the detailed date includes temperature at 5% weight loss (T_5%_), temperature for the maximum decomposition rate (T_max_), and char residues at 600 °C, as shown in Table 5. The degradation of the pure PVA and MXene/PVA nanocomposites without any PVPA or PEPA presented four weight loss stages during the heating process, and the main decomposition process began from 250 °C to 450 °C.

It can be seen from Table 5 that T_5%_ of MXene/PVA nanocomposites improved, but that of PVA containing PVPA and PEPA dropped when compared with the pure PVA, and MXene also ameliorated T_5%_ of MXene/PVA nanocomposites with PVPA and PEPA. The weight loss before about 150 °C was the first decomposition stage, and as the free water was small in PVA, MXene, PVPA, and PEPA, it can be easily volatilized in this stage.

The second stage temperature ranged from about 150 °C to about 350 °C, and the mass loss rate increased when the temperature reached above 250 °C, as shown in Figure 9. At this time, PVPA and PEPA can form the inorganic residue and carbonaceous char layer on the surface of the nanocomposites. The third stage corresponds to the elimination of PVA molecular side groups and molecular chain breakage and degradation of the PVA [32], PEPA, and PVPA. MXene undergoing thermal oxidation should also be included. The temperature of the last stage is about over 450 °C, MXene can improve the char residue yield at 600 °C. Moreover, the weight-loss rates of MXene/PVA nanocomposites were lower than that of PVA. These enhancements for MXene/PVA nanocomposites containing both PVPA and PEPA, or not, are due to the existence of MXene, which can improve the thermal stability of the PVA matrix.

The initial decomposition temperature and temperature for the maximum decomposition rate of the MXene/PVA nanocomposites decreased slightly as the loading of MXene increased, as presented in Figure 9, corresponding to the high thermal conductivity of MXene which can reduce the thermal decomposition temperature of the nanocomposite. MXene can accelerate the formation of a char layer on the surface of the nanocomposite, which provides better protection to PVA. It seems that the addition of PVPA and PEPA together in the PVA matrix weakened the thermal stability of PVA due to the mechanism action of the novel intumescent fire retardants.

**Figure 10:**
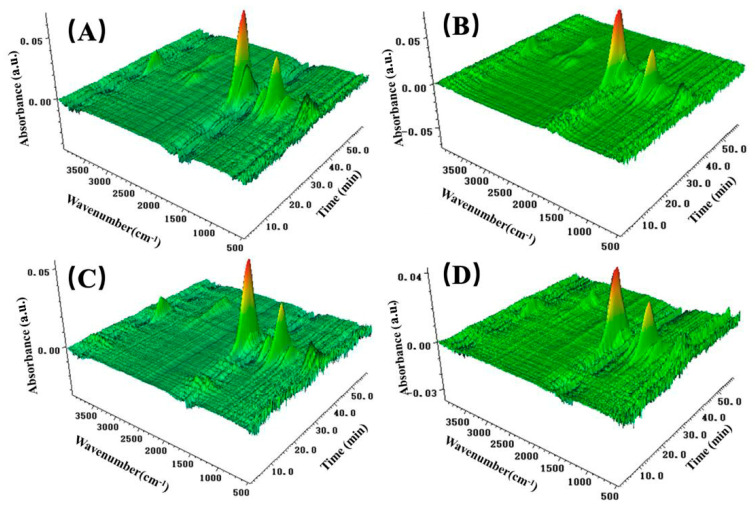
3D TG-FTIR spectra of the gaseous products of PVA and MXene/PVA nanocomposites: (**A**) PVA; (**B**) MXene_1.0%_/PVA; (**C**) PVA-PVPA_10%_-PEPA_5%_ and (**D**) MXene_1.0%_/PVA-PVPA_10%_-PEPA_5%_.

**Figure 11:**
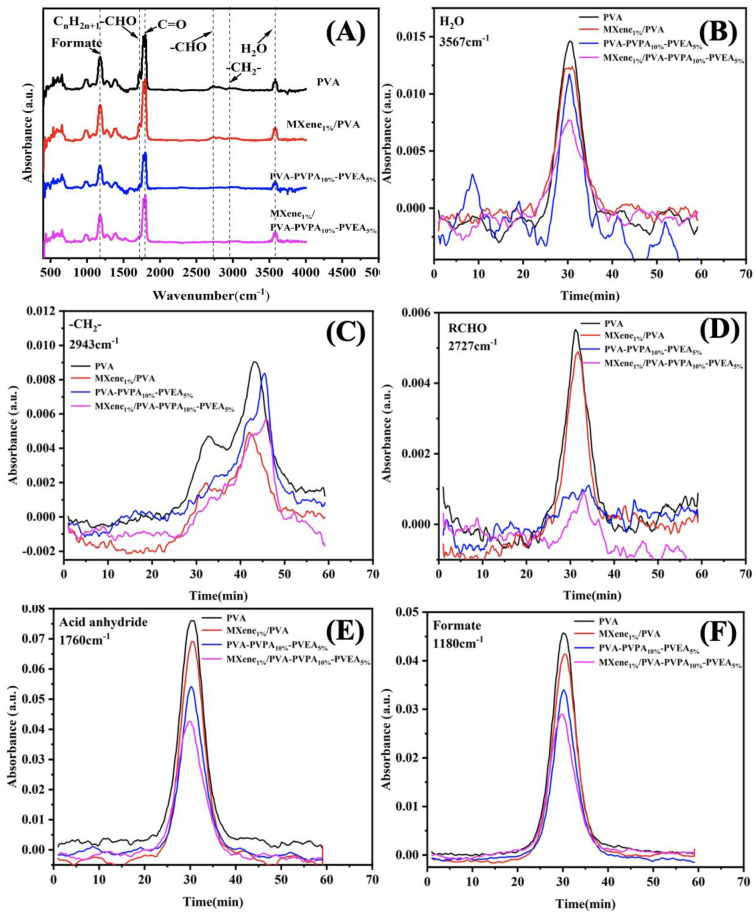
FTIR spectra (**A**) of gaseous pyrolysis products for PVA and MXene/PVA nanocomposites at the maximum evolution rate; Intensities of characteristic peaks for pyrolysis products of PVA and MXene/PVA nanocomposites (**B**–**F**).

The TG-FTIR test was utilized to investigate the volatile species of the PVA and MXene/PVA nanocomposites during the thermal decomposition process. Three-dimensional TG-FTIR spectra of the gaseous products of the PVA and MXene/PVA nanocomposites are presented in Figure 10. The FTIR of gaseous pyrolysis products for PVA and MXene/PVA nanocomposites is illustrated in Figure 11A, which was obtained at the maximum evolution rate during the thermal degradation. The intensities of characteristic peaks for pyrolysis products of PVA and MXene/PVA nanocomposites are shown in Figure 11B–F.

The gaseous pyrolysis products are identified by the characteristic of strong FTIR signals, such as H_2_O (3567 cm^−1^), hydrocarbons (2943 cm^−1^), aldehydes (2727 cm^−1^), acid anhydride (1760 cm^−1^), and formate (1180 cm^−1^) [33,34]. The absorption peaks of the pyrolysis products of PVA are similar to those of MXene/PVA nanocomposites, which implies that the addition of MXenne, PVPA, and PEPA in PVA does not obviously change the thermal degradation of the PVA matrix. As shown in Figure 11B–F, the absorption intensities of pyrolysis products of PVA containing PVPA and PEPA are weaker than that of pure PVA. Moreover, the addition of MXene in PVA with or without PVPA and PEPA can lower the absorption intensities of pyrolysis products from the PVA matrix. That can be attributed to the MXene with a 2D layered structure, which can be regarded as a physical barrier to delay and reduce the escape of pyrolysis products from substrates. MXene can effectively inhibit gas diffusion and protect the PVA from heat and flame.

### 3.4. Mechanical Properties

**Figure 12:**
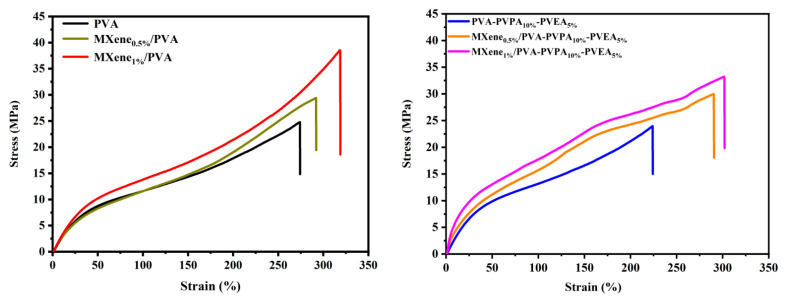
Stress–strain curves of PVA and MXene/PVA nanocomposites.

The stress–strain of curves of PVA and MXene/PVA nanocomposites are illustrated in Figure 12, and the tensile strength, Young’s modulus, and elongation at break of PVA and MXene/PVA nanocomposites are presented in Table 6. It should be noted from Figure 12 and Table 6 that the tensile strength and Young’s modulus of MXene/PVA nanocomposites with or without PVPA and PVEA have increased with the increase in MXene contents in the PVA matrix. Tensile strength has increased by approximately 20.0% to 62.9%. while the Young’s modulus also was greatly improved when compared to the PVA matrix, because the addition of MXene nanosheets can distribute the load on the polymer chain and play a certain role in carrying the load. The nano-constrained structure formed by hydrogen bonding hinders the mobility of polymer chains, which greatly helps to improve the tensile properties of nanocomposites, and it also leads to an increase in the tensile modulus [35].

The elongation at break of the MXene/PVA nanocomposites, which contains PVPA and PVEA or not, have also improved. The elongation at break reached the peak for the MXene/PVA nanocomposite with only 1.0 wt.% of MXene, which was 318.8%, and it was up by 16.3% compared to that of the pure PVA. Furthermore, PVA-PVPA_15%_ has a break elongation of 162.9%, whereas MXene_1.0%_/PVA-PVPA_10%_-PVEA_5%_ has a break elongation of 301.9%. The reason lies in the abundant functional groups including the –OH, –F, and O which can be good integration with PVA, and the interfacial interaction between MXene and the PVA matrix was further enhanced. These factors contribute to the formation of the rigid network in the system, and can significantly improve mechanical properties. In addition, it can be noted that the elongation at break of the pure PVA would be decreased after the addition of PVPA and PVEA. The true cause lay in the fact that phosphonic acid can have the chemical reaction with the –OH in PVA that made the structure of the matrix more compact [36].

## 4. Conclusions

In this work, the flame-retardant properties, thermal stability, and mechanical properties of MXene/PVA nanocomposites containing PVPA and PEPA were investigated. MXene has similar flame-retardant properties to PVA because the MXene covered the surface of the char layer, isolating the air to achieve good fire resistance. Moreover, PVPA and PEPA further improved the flame-retardant properties of MXene/PVA nanocomposites. PVPA and PEPA do not obviously change the thermal degradation of the MXene/PVA matrix. In addition, MXene improved the thermal stability of the PVA matrix. The tensile strength and Young’s modulus of MXene/PVA nanocomposites increased with the increase in MXene content in the PVA matrix. The elongation at the break of MXene/PVA nanocomposites reached a maximum when the MXene mass fraction was 1.0 wt.%, regardless of whether PVPA and PEPA were present in the PVA matrix.

## Figures and Tables

**Table 1:** Formulation of MXene/PVA nanocomposites.

Samples	PVA (wt%)	PVPA (wt%)	PEPA (wt%)	Ti_3_C_2_T*_x_* (wt%)
PVA	100.0	0.0	0.0	0.0
MXene_0.5%_/PVA	99.5	0.0	0.0	0.5
MXene_1.0%_/PVA	99.0	0.0	0.0	1.0
PVA-PVPA_10%_-PEPA_5%_	85.0	10.0	5.0	0.0
MXene_0.5%_/PVA-PVPA_10%_-PEPA_5%_	84.5	10.0	5.0	0.5
MXene_1.0%_/PVA-PVPA_10%_-PEPA_5%_	84.0	10.0	5.0	1.0

**Table 2:** LOI and UL94 test results of PVA and MXene/PVA nanocomposites.

Samples	LOI Value (%)	UL 94		
		Rating	Dripping	Ignition of Cotton
PVA	19.00 ± 0.19	V2	Yes	Yes
MXene_0.5%_/PVA	19.50 ± 0.24	V2	Yes	Yes
MXene_1.0%_/PVA	21.00 ± 0.28	V1	Yes	No
PVA-PVPA_10%_-PEPA_5%_	25.50 ± 0.33	V0	No	No
MXene_0.5%_/PVA-PVPA_10%_-PEPA_5%_	26.00 ± 0.38	V0	No	No
MXene_1.0%_/PVA-PVPA_10%_-PEPA_5%_	27.50 ± 0.44	V0	No	No

**Table 3:** Cone calorimetry data of PVA and MXene/PVA nanocomposites.

Samples	TTI (s)	pHRR (kW/m^2^)	FPI (s·m^2^/kW)	THR (MJ/m^2^)	TSR (m^2^/m^2^)	Residue Mass (%)
PVA	9	1092 ± 3	0.008 ± 0.0002	16.7 ± 0.1	287.5 ± 0.3	0.3 ± 0.1
MXene_1.0%_/PVA	10	824 ± 3	0.012 ± 0.0001	16.5 ± 0.1	240.4 ± 0.4	4.1 ± 0.1
PVA-PVPA_10%_-PEPA_5%_	12	721 ± 6	0.017 ± 0.0004	16.1 ± 0.2	216.9 ± 0.7	8.1 ± 0.2
MXene_1.0%_/PVA-PVPA_10%_-PEPA_5%_	14	755 ± 7	0.019 ± 0.0005	12.4 ± 0.1	134.6 ± 0.8	9.5 ± 0.2

**Table 4:** Element composition of the char residue of PVA and MXene/PVA nanocomposites after cone calorimeter test.

Specimens		C	O	Ti	P	N
PVA	Weight/%	82.73	17.27	/	/	/
	Atmoic/%	86.45	13.35	/	/	/
	Error/%	9.48	2.47	/	/	/
MXene_1.0%_/PVA	Weight/%	60.14	16.16	23.70	/	/
	Atmoic/%	76.89	15.51	7.60	/	/
	Error/%	6.94	2.28	4.19	/	/
PVA-PVPA_10%_-PEPA_5%_	Weight/%	52.56	29.32	/	13.23	4.89
	Atmoic/%	62.65	26.23	/	6.11	5.00
	Error/%	3.98	2.36	/	0.40	0.60
MXene_1.0%_/PVA-PVPA_10%_-PEPA_5%_	Weight/%	67.68	10.87	15.06	6.39	/
	Atmoic/%	82.44	9.94	4.60	3.02	/
	Error/%	7.69	1.57	2.89	0.32	/

**Table 5:** TGA data of the PVA and MXene/PVA nanocomposites under nitrogen atmosphere.

Samples	T_5%_ (°C)	T_max1_ (°C)	Δ_weight1_ (%)	T_max2_ (°C)	Δ_weight2_ (%)	T_max3_ (°C)	Δ_weight3_ (%)	Char Residue at 600 °C (%)
PVA	159.6	150.1	7.84	349.6	62.2	452.6	26.06	3.90
MXene_0.5%_/PVA	160.4	151.4	7.76	350.4	61.99	453.4	24.58	4.39
MXene_1.0%_/PVA	169.9	154.9	7.56	352.4	61.14	455.4	26.90	5.67
PVA-PVPA_10%_-PEPA_5%_	146.8	149.3	10.51	352.3	50.68	459.3	30.91	7.90
MXene_0.5%_/PVA-PVPA_10%_-PEPA_5%_	159.6	152.1	14.75	334.6	56.67	460.1	20.39	8.19
MXene_1.0%_/PVA-PVPA_10%_-PEPA_5%_	170.5	173.1	9.90	340.5	55.36	462.5	25.56	9.17

Note: Δ_weight1_, Δ_weight2_, and Δ_weight3_ are the weight-loss percentage at T_max1_, T_max2_, and T_max3_, respectively.

**Table 6:** The tensile strength and elongation at break of PVA and MXene/PVA nanocomposites.

Samples	Elongation at Break (%)	Strength (MPa)	Young’s Modulus (GPa)
PVA	274.2 ± 3.5	24.8 ± 0.8	32.8 ± 0.3
MXene_0.5%_/PVA	292.0 ± 4.3	29.4 ± 1.3	35.2 ± 0.8
MXene_1.0%_/PVA	318.8 ± 4.7	38.2 ± 0.9	39.1 ± 1.1
PVA-PVPA_10%_-PVEA_5%_	224.2 ± 5.2	24.0 ± 3.6	40.5 ± 3.4
MXene_0.5%_/PVA-PVPA_10%_-PVEA_5%_	290.5 ± 5.4	30.0 ± 3.4	68.8 ± 5.1
MXene_1.0%_/PVA-PVPA_10%_-PVEA_5%_	301.9 ± 4.8	33.2 ± 2.7	92.2 ± 7.8

## Data Availability

Not applicable.
